# Case Report: Destructive deep infection of the foot caused by *Neoscytalidium dimidiatum* leading to great toe amputation

**DOI:** 10.3389/fimmu.2026.1791879

**Published:** 2026-03-05

**Authors:** Huiyu Wang, Yiyun Xu, Hong Li, Ying Chen, Zuocai Wang, Zhenlong Shang, Menglan Zhou, Yingchun Xu

**Affiliations:** 1 Department of Clinical Laboratory, Qionghai People’s Hospital, Qionghai, China; 2 Department of Laboratory Medicine, Peking Union Medical College Hospital, Chinese Academy of Medical Sciences & Peking Union Medical College, Beijing, China; 3 Graduate School, Chinese Academy of Medical Sciences & Peking Union Medical College, Beijing, China; 4 Department of Pathology, Qionghai People’s Hospital, Qionghai, China; 5 Department of Trauma Surgery, Qionghai People’s Hospital, Qionghai, China

**Keywords:** dematiaceous mold, diabetic foot infection, invasive fungal infection, *Neoscytalidium dimidiatum*, toe amputation

## Abstract

We report an invasive deep-tissue infection caused by *Neoscytalidium dimidiatum* in a diabetic patient in China. Although typically a phytopathogen restricted to superficial human infections, it caused rapid necrosis necessitating hallux amputation. This case highlights the need for clinical awareness regarding the pathogenic potential of *N. dimidiatum* in immunocompromised hosts.

## Introduction

*Neoscytalidium dimidiatum* is a rapidly growing dematiaceous mold that is widely encountered in soil and decaying vegetation in tropical and subtropical environments. Although being a common phytopathogen, it has increasingly been recognized as an emerging non-dermatophyte cause of superficial human infection involving the skin and nails (1). Invasive deep tissue infection is uncommon, but when it occurs, the clinical course is often aggressive and typically requires a combination of systemic antifungal therapy and surgical management. In non-endemic region, limited awareness of this organism may lead to underestimation of its clinical significance and delay in appropriate diagnosis and treatment.

## Case presentation

A 72-year-old man with more than 10-year history of poorly controlled type 2 diabetes presented with a right plantar soft-tissue infection manifested by redness, pain and scant purulent discharge after stepping on a nail, without fever or systemic symptoms.

He reported a peak blood glucose level of 19 mmol/L and had been managed with metformin plus premixed insulin aspart 30 (8 IU twice daily). However, treatment adherence was poor, and he did not perform routine glycemic monitoring. Initial treatment at a local clinic was ineffective, and he was admitted with severe soft-tissue infection of the right foot.

On admission, physical examination revealed a 0.5×0.5 cm plantar ulcer with tenderness and local warmth, accompanied by diminished sensation of the right hallux. Laboratory test indicated significantly elevated inflammatory markers: blood glucose 7.47 mmol/L, glycated hemoglobin 9.00%, white blood cell counts 20.84×10^9^/L, hemoglobin 126 g/L, neutrophil percentage 85.2%, interleukin-6 41.58 pg/mL, C-reactive protein133.64 mg/L, and procalcitonin 0.13 ng/L. After admission, he was started on insulin aspart (NovoRapid) 8 IU every 8 hours plus insulin glargine 10 IU once daily, with regular bedside glucose monitoring; fasting plasma glucose decreased to 5.5 mmol/L after 3 days. Initial wound secretion cultures grew *Klebsiella pneumoniae, Enterococcus faecalis, and Serratia odorifera.* Although treatment with piperacillin/tazobactam and regular dressing changes showed some efficacy, the wound condition failed to improve.

**Figure 1:**
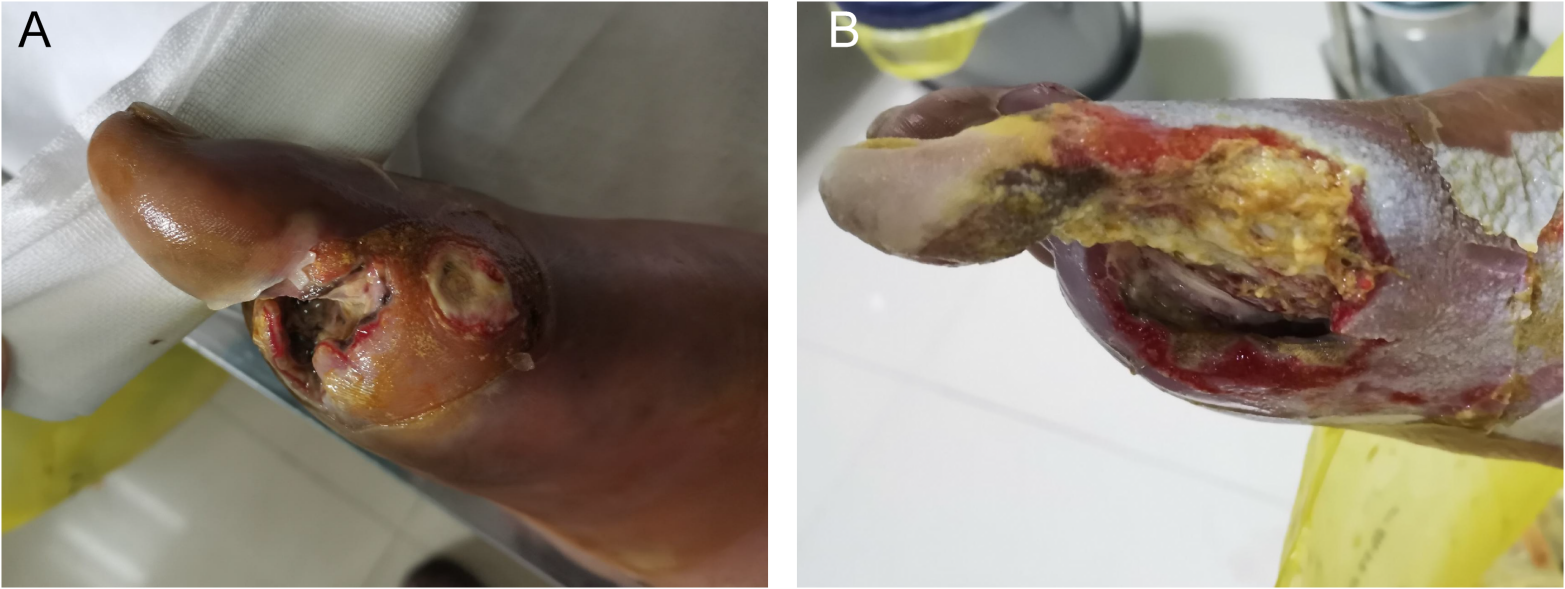
Ulceration and debridement of the infected right foot. **(A)** Pre-debridement presentation showing tissue ulceration and necrosis. **(B)** Post-operative view following the initial surgical debridement and excision of necrotic tissue.

**Figure 2:**
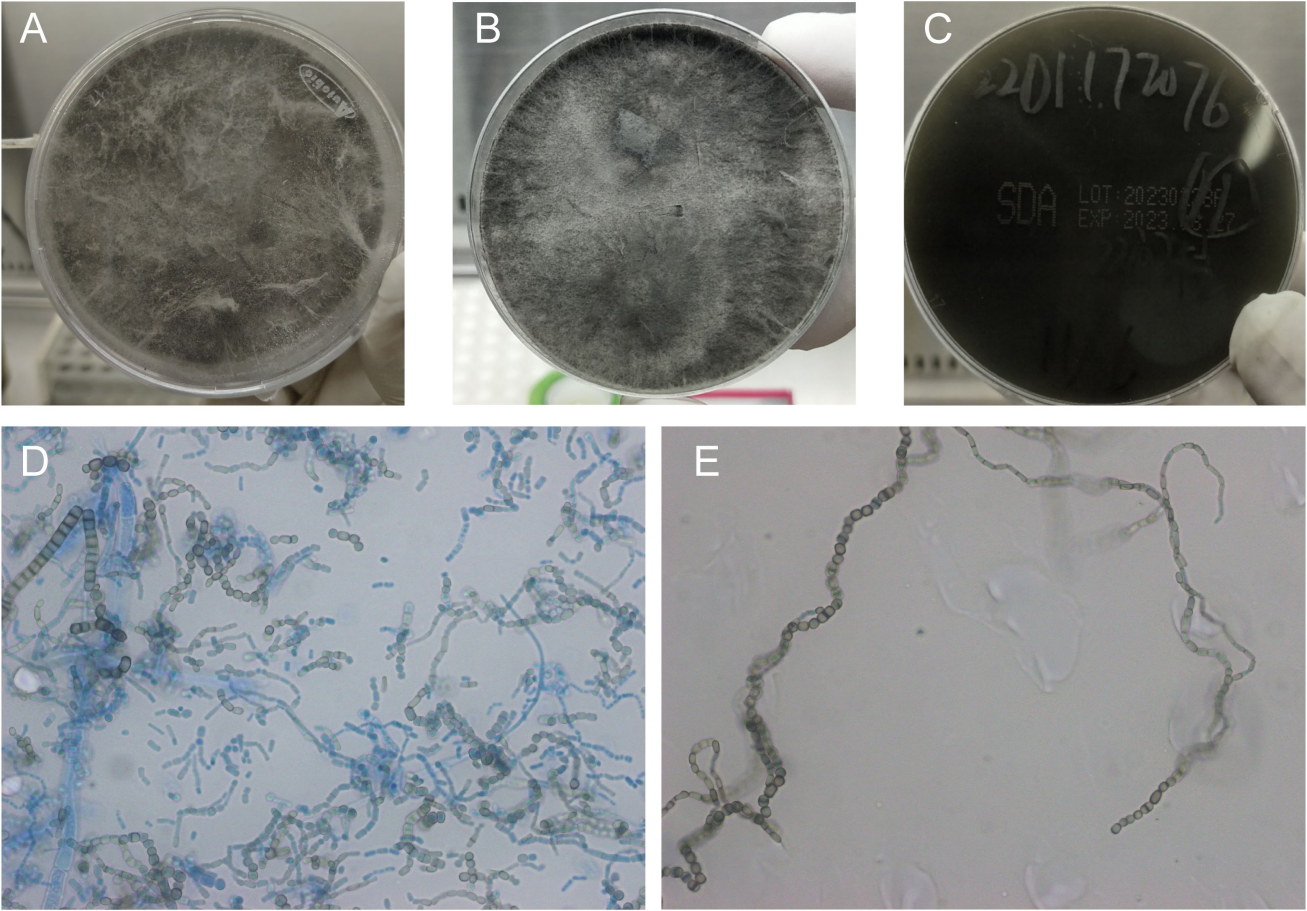
Colony morphology of the isolated *N. dimidiatum*. **(A)** Colony presentation on SDA after 5 days of incubation at 35 °C. **(B, C)** Obverse and reverse views of the colony after 7 days of incubation on SDA at 35 °C. **(D, E)** Microscopic morphology visualized with lactophenol cotton blue staining after 7 days of culture on SDA at 35 °C (original magnification ×1000).

On hospital day 5, the wound had expanded to 3cm×4cm (**Figure 1A**). Surgical debridement and vacuum sealing drainage (VSD) were performed (**Figure 1B**). Intraoperative findings revealed necrosis and ulceration of the plantar skin and subcutaneous tissue, accompanied by a foul odor and compromised local perfusion. Deep tissue samples were collected intraoperatively; homogenized tissues were inoculated onto blood agar and chocolate agar, while non-homogenized tissue blocks were inoculated directly onto Sabouraud Dextrose Agar (SDA). No bacterial growth was observed on blood or chocolate agar, but filamentous fungi were noted. The fungal colonies on SDA grew rapidly, initially presenting as white, radial colonies with sparse aerial hyphae and a pale-yellow reverse. After five days, the colonies filled the plate, transitioning from white to grayish brown (**Figure 2A**). Two more days later, the colonies had turned black (**Figures 2B, C**). Lactophenol cotton blue staining revealed branched, septate, broad, brown hyphae producing arthroconidia. The brown arthroconidia appeared round, oval, cylindrical, or barrel-shaped and were arranged in long chains (**Figures 2D, E**). Internal transcribed spacer (ITS) sequencing demonstrated 100% similarity to *N. dimidiatum* (GenBank No.MH251955.1).

**Figure 3:**
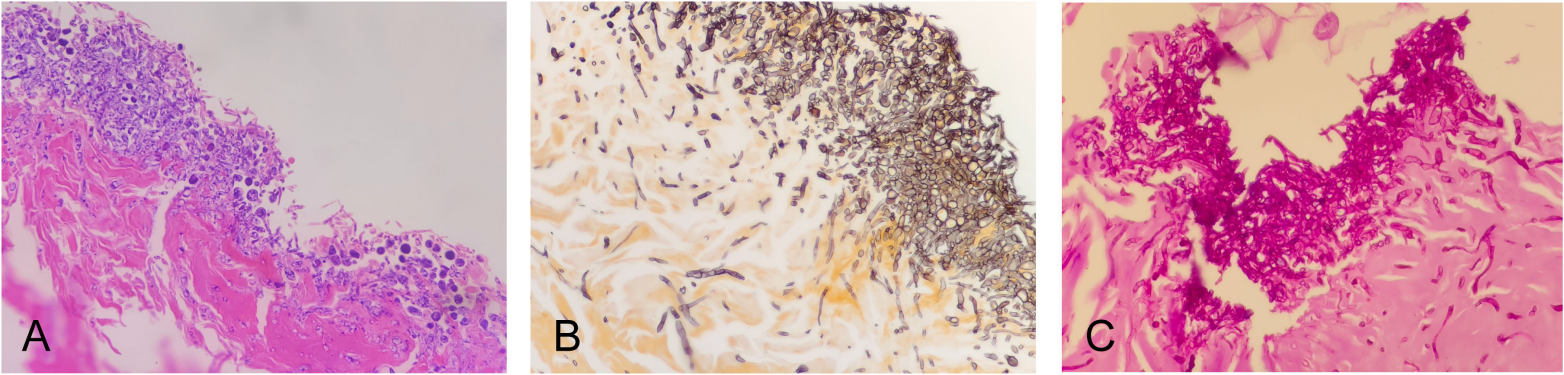
Histopathological examination of the necrotic tissue from the intraoperative site. **(A)** H&E staining revealing degeneration, necrosis, inflammatory cell infiltration, and abundant septate hyphae. **(B)** GMS staining showing numerous brownish black septate hyphae. **(C)** PAS staining demonstrating abundant pink septate hyphae. (original magnification ×400).

Despite continuous postoperative VSD therapy, the wound progressively enlarged to 6cm×5cm, accompanied by blackening and necrosis of the right hallux. By hospital day 18, the wound surface area had extended to 6cm×7cm. Consequently, the patient underwent repeat debridement and right hallux amputation, with tissue culture still growing *N. dimidiatum*. Hematoxylin and Eosin (H&E) staining of the operative tissue revealed degeneration, necrosis, inflammatory cell infiltration, and septate hyphae (**Figure 3A**). Grocott’s methenamine silver (GMS) staining showed abundant brownish-black septate hyphae (**Figure 3B**), and Periodic Acid-Schiff (PAS) staining revealed numerous red septate hyphae (**Figure 3C**).

*In vitro* antifungal susceptibility testing revealed low minimum inhibitory concentrations (MICs) against nearly all the drugs tested excepted for flucytosine (MIC = 2) and fluconazole (MIC = 4) (**Table 1**). The clinical team recommended voriconazole therapy and emphasized that untreated invasive fungal infection could lead to severe complications, including progressive tissue destruction and systemic dissemination. Despite this, the patient declined antifungal treatment due to personal concerns and financial constraints, which limited therapeutic options and ultimately contributed to disease progression and the need for amputation. Fortunately, the conditions gradually improved with continued VSD and dressing change after amputation. Follow-up visit at 1 and 3 months showed good wound healing with no recurrence.

**Table 1:** *In vitro* antifungal susceptibility profile of the *Neoscytalidium dimidiatum* isolate.

Antifungal agent	*Neoscytalidium dimidiatum*	
	MIC	interpretation
Amphotericin B	1	NA
Flucytosine	2	NA
Fluconazole	4	NA
Voriconazole	0.06	NA
Itraconazole	0.5	NA
Isavuconazole	0.25	NA
Posaconazole	0.5	NA
Micafungin	0.06	NA
Caspofungin	0.015	NA

NA, not available. Current CLSI and EUCAST guidelines have not established clinical breakpoints or interpretive criteria for *N. dimidiatum*. Therefore, S/I/R categorization is not provided in this table, and the MIC results are reported for *in vitro* activity reference only.

## Discussion

*N. dimidiatum* is primarily a phytopathogen but is increasingly recognized as an opportunistic human pathogen. It most commonly causes superficial infections involving the plantar skin, interdigital spaces and toenails, typically resembling tinea pedis or onychomycosis and manifesting as plantar scaling and nail thickening with onycholysis (2, 3). In China, human infections have also been documented. For instance, Yang et al. described two cases of deep foot infection caused by *N. dimidiatum* in Taiwan, presenting as cellulitis with abscesses formation. Clinical improvement was achieved only after intravenous amphotericin B therapy (4). In addition, a case series of mycetoma in southern India identified *N. dimidiatum* as a rare cause of eumycetoma, with the foot and lower extremities being the most affected sites (5). These reports underscore the potential for this fungus to establish deep, focal, chronic infections likely following traumatic inoculation. These findings align with prior reports of invasive disease in immunocompromised hosts (such as those with liver cirrhosis, organ transplants, or diabetes), demonstrating that *N. dimidiatum* can breach superficial barriers and disseminate to deep-seated structures (6–8). Notably, in contrast to the Taiwanese cases, our case presented in the context of a typical diabetic foot. Despite polymicrobial wound cultures and antibacterial therapy, the infection progressed rapidly with extensive tissue necrosis, ultimately necessitating hallux amputation. This clinical course suggests that *N. dimidiatum* may exhibit enhanced focal invasiveness and tissue destructive potential in the setting of diabetic foot disease.

Epidemiologically, the patient had a history of agricultural exposure prior to admission, suggesting that contaminated soil or infected plant matter served as the primary source of transmission. Furthermore, the patient’s poorly controlled diabetes contributed to a compromised immune status. It also suggested that the hyperglycemic microenvironment provides an optimal carbon source for fungal proliferation, thereby facilitating the progression of this invasive infection (9).

A critical diagnostic challenge lies in the potential for bacterial co-infections to mask deep-seated fungal pathogens. In the present case, initial wound cultures indicated a polymicrobial bacterial infection, a finding that could easily divert clinical attention from a potential fungal etiology. Consequently, when standard antibacterial regimens prove ineffective and necrosis progresses, clinicians should maintain a high alert of suspicion for non-bacterial pathogen and promptly obtain deep tissue specimens for definitive diagnosis.

Currently, there is no standardized therapeutic regimen for *N. dimidiatum* infection, and management often relies on empirical therapy. Previous literature suggests that amphotericin B or voriconazole are commonly employed for deep infections (6–8, 10). The *N. dimidiatum* isolate in this case demonstrated low MICs for both amphotericin B and voriconazole, prompting a clinical recommendation for voriconazole therapy. However, due to the patient’s refusal of antifungal therapy, the *in vivo* efficacy of these agents remained unverified in this patient. During hospitalization, the patient was clearly informed that, in the context of immunodeficiency, a deep fungal infection without appropriate systemic antifungal therapy carries a significant risk of recurrence and may ultimately necessitate repeat amputation. Despite this counseling, the patient declined systemic antifungal treatment due to financial limitations. Consequently, the original management strategy, comprising VSD and regular dressing changes—was continued. Fortunately, following the hallux amputation, adequate surgical management facilitated favorable wound healing without signs of persistent *N. dimidiatum* infection. This outcome underscores the crucial role of aggressive surgical intervention in controlling the progression of deep-seated mold infections.

In conclusion, this case highlights the invasive potential of *N. dimidiatum* in deep-seated tissues, offering critical insights into the diagnosis and management of complex diabetic foot infections. Furthermore, it underscores the vital importance of deep tissue sampling and dedicated fungal culture, while reaffirming the indispensable role of surgical debridement in the successful management of complex fungal infections.

## Data Availability

The raw data supporting the conclusions of this article will be made available by the authors, without undue reservation.

## Supplementary material

Supplementary Figure 1 Clinical timeline of the diabetic foot infection caused by *N. dimidiatum.* TZP, piperacillin/tazobactam; VSD, vacuum sealing drainage. TZP, piperacillin/tazobactam; VSD, vacuum sealing drainage.
