# Supplementary figures and images for: Case Report: Destructive deep infection of the foot caused by Neoscytalidium dimidiatum leading to great toe amputation
Source: Front Immunol. 2026 Mar 5;17:1791879. doi: 10.3389/fimmu.2026.1791879 (PMC12999427; doi:10.3389/fimmu.2026.1791879)

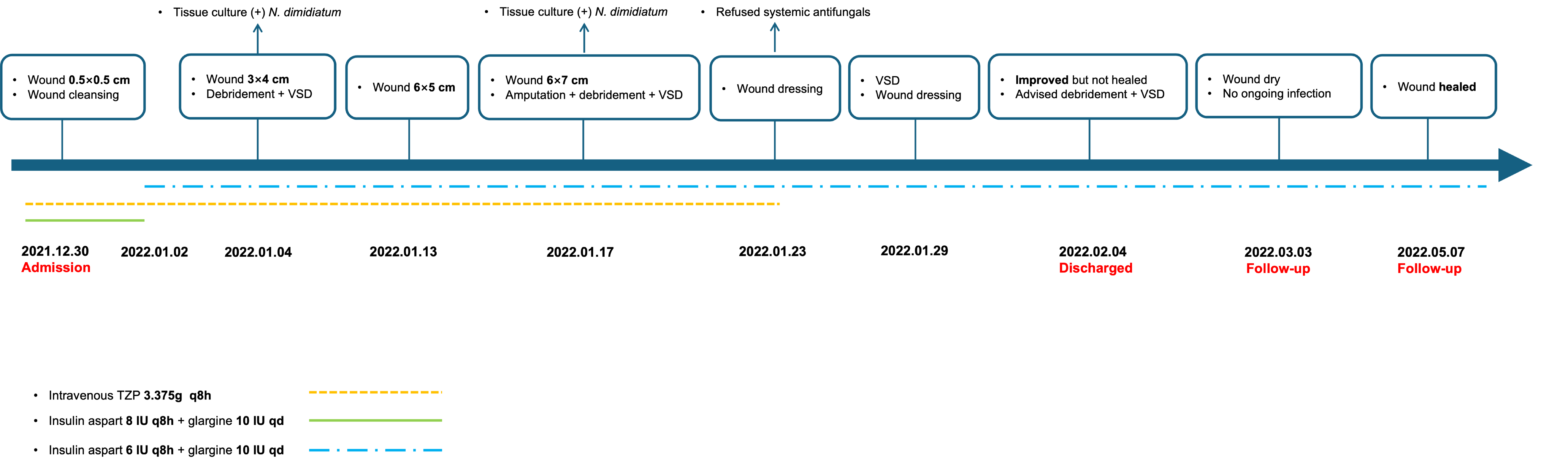

Supplement: Supplementary Figure 1 — Clinical timeline of the diabetic foot infection caused by N. dimidiatum. TZP, piperacillin/tazobactam; VSD, vacuum sealing drainage. TZP, piperacillin/tazobactam; VSD, vacuum sealing drainage. [file Image1.tif]
